# 

**Published:** 1834-10-01

**Authors:** 


### BIBLIOGRAPHICAL RECORD;

OR,

*Works received for Review since last Quarter.*

1. A new system of Organic Chemistry, translated from the French of F. V. Raspail, with Notes and Additions. By WILLIAM HENDERSON, M. D. Lecturer on Materia Medica in the University of Aberdeen. 8vo. pp. 602, with numerous plates. June, 1834.

2. A practical Treatise on the Diseases of the Uterus and its Appendages; translated from the French of Madame Veuve Boivin, Sage-femme Surveillante en Chef de la Maison de Santé, &c. and A. Dugés, Professeur a la Faculté de Medecine de Montpellier, with copious Notes. By G. O. HEMING, F.L.S. Consulting Accoucheur to the St. Pancras Infirmary, &c. Two volumes 8vo (one of them plates), pp. 560. Lond. 1834. 14s. and 12s. boards.

3. Anatomie Pathologique du Corps Humain, &c. Par M. CRUVEILHIER. 19th Livraison, Maladies du Foie, de la Rate, et du Grand Epiploon, &c. Bailliere, July, 1834.

4. Chemical Recreations:—a Series of amusing and instructive Experiments, which may be performed with ease, safety, success and economy. To which is added, The Romance of Chemistry, an Inquiry into the Fallacies of the prevailing Theory of Chemistry, &c. By JOHN JOSEPH GRIFFIN. Small 8vo. pp. 372, with numerous woodcuts; 7th edition, entirely re-written. Glasgow, June, 1834.

5. A practical Treatise on Medical Jurisprudence, with so much of Anatomy, Physiology, Pathology, and the Practice of Medicine, as are essential to be known by Members of Parliament, Lawyers, Coroners, Magistrates, Officers in the Army and Navy, and private Gentlemen; and all the Laws relating to Medical Practitioners: with explanatory plates. By J. CHITTY, Esq. Barrister at Law. Part I. royal 8vo, pp. 466, with Index, price One Guinea. Butterworth, London, July, 1834.

6. The Surgical and Descriptive Anatomy of the Bones, Ligaments, and Joints. By W. H. THOMAS, M.R.C.S. Small 8vo, pp. 309. Longman & Co. July, 1834. Price 6s.

7. The Liverpool Medical Journal; published monthly, under the superintendence of an Association of Physicians and Surgeons, &c. Nos. 3 & 4, for July and August, 1834. *In exchange.*

8. L'Observateur Medical Belge; Journal de Medecine, &c. Avril et Mai, 1834. *In exchange.*

9. Clinical Lectures in the Manchester Infirmary. By EDWARD CARBUTT, M.D. 1 vol. 8vo, pp. 407. Longman & Co. July 1834.

10. Illustrations of the Natural History of Worcestershire, with information on the Statistics, Zoology and Geology of the County; including also, A short Account of the Mineral Waters. By CHARLES HASTINGS, M.D. 8vo. pp. 181, with Appendices, Maps, &c. Sherwood and Co. July, 1834.

11. An Essay on the Relation of the Theory of Morals to Insanity. By T. MAYO, Fellow of the Royal College of Physicians.

12. Some Observations on the Preparation and Medicinal Employment of the Ioduret and Hydriodate of Iron. By A. T. THOMSON, M.D. &c. 8vo. pp. 64, sewed. July, 1834.

13. A Synoptical View of the Diseases of the Chest, as illustrated either by Auscultation, Percussion, Thoracic Measurement, and Succussion; or by other Sources of Information. By RICHARD MADDOCK HAWLEY, M.D. Fellow of the Royal College of Physicians, Ed. &c. Atlas, pp. 6. 1834.

☞ *This is the most scientific and accurate auscultic Chart that has yet appeared. It will prove very useful to stethoscopic students and practitioners.*

14. The Principles of Physiology applied to the Preservation of Health, &c. &c. By AND. COMBE, M.D. Second edit. enlarged. 1834.

☞ *We are very glad to observe that the first edition was exhausted in three or four months. This edition is greatly improved.*

---

15. A Dictionary of Terms, employed by the French, in Anatomy, Physiology, Pathology, Practice, &c. with their derivations from the Greek and Latin, their Synonimes, &c. By SHIRLEY PALMER, M.D. Part I. pp. (no pages), royal 8vo, double column. July, 1834. Price 6s.

---

16. Reflections on the Nature of Inflammation, and its alleged Consequences. By DAVID BADHAM, M.D. one of Dr. Radcliff's Travelling-fellows from the University of Oxford. 8vo, pp. 67. Glasgow, July 1834.

---

17. Fragmenta de Viribus Medicamentorum positivis, sive in Sano Corpore Humano observatis. A SAMUELE HAHNEMAN, M.D. &c. Edidit F. F. QUIN, M.D. Medicus Ordinarius Leopoldi Primi Regis Belgarum, &c. 8vo, pp. 214. Londini, 1834.

---

18. A Series of Anatomical Plates, in Lithography, &c. Edited by JONES QUAIN, M.D. Professor of Anatomy and Physiology in the University of London. Division I. —Muscles. Fasciculi I. to XIV. Price Two Shillings each Fasciculus.

☞ *We have felt deep regret at being compelled, on various occasions, to speak unfavourably of the execution of these plates. We are happy to say, that the two or three last fasciculi display a very decided improvement. The character of Mr. Quain, and the prominent and honourable station which he occupies, should make him most careful not to permit inferior productions to receive the sanction of his name. An active superintendence of the draughtsman is indispensable on his part, and the lithographist should remember, that clearness and distinctness, in the shading and the outline, are absolutely necessary, in what must be not only drawings, but maps. It shovld be the aim of all parties concerned, to publish a set of plates superior to the common run of their predecessors.*

*We would strongly recommend Mr. Quain to abandon the idea of mixing up physiological comments with a description of the plates. What, in the name of Common Sense, has physiology to do with such an undertaking? The purchaser of the plate considers it a most intolerable nuisance, to be forced to wade through pages of irrelevant discussion, before he can arrive at the matter of fact, the description, and elucidation of the drawings placed before him. We repeat, that we perceive an improvement in the last fasciculi. We shall watch the progress of the work, and we hope to speak of it more favourably as it proceeds.*

---

19. An Explanation of the Causes why Vaccination has sometimes failed to prevent Small-Pox, and also a Description of a Method, confirmed by Experience, of obviating such Causes. By EDWARD LEESE, &c. Part the Second. Octavo, stitched, pp. 36. London, 1833.

☞ *An interesting pamphlet, from a zealous and amiable man.*

---

20. A Collection of Geological Facts and Observations, intended to elucidate the Formation of the Ashby Coal-field, in the parish of Ashby-de-la-Zouch, and the neighbouring District; being the Result of Forty Years' Experience. By EDWARD MAMMATT, F.G.S. Illustrated by a Map and Profiles, coloured Sections of the Stratification, and 102 Plates of Vegetable Fossils, after Drawings taken from Nature. Royal 4to. Ashby-de-la-Zouch and London, 1834.

☞ *This Work of Mr. M.'s must necessarily exert important influences on the study of Geology, in contributing, by* FACTS, *to establish and extend the fundamental principles of this most interesting science, and in tending, by* OBSERVATIONS, *to restrain that wild propensity to fabricate theories by which its progress and usefulness have too long been injuriously confined. For these reasons, we would prefer its claims for approbation on the attention of our geological readers. Chiefly, however, we bring Mr. M.'s instructive pages under the notice of physicians and other cultivators of the healing art, on the ground that it communicates a diversity of valuable information, on the Temperature of Mines and its bearings on the subject of a 'Central Heat,' on the formation of Thermal Springs, on the evolution of Carburetted Hydrogen Gas, on the uses of a Fossil Botany, and especially on the Nature and Properties of the Bromated Mineral Waters of Ashby-de-la-Zouch, and their peculiar virtues in preventing the development of the Scrofulous and Consumptive Predispositions, as well as their energetic agency in perfecting the treatment of many inveterate chronic diseases.*

21. Observations on the Preservation of Hearing, and on the Choice, Use and Abuse of Ear-trumpets, &c. By J. H. CURTIS, Esq. Aurist to the King.

22. Oratio Harveiana in Novis Ædibus Collegii habita sext. kalend. Jul. an. MD CCCXXXIV. Ab EDVARDO THOMA MONRO, M.D. Col. Reg. Med. Lond. Socio. Londini, MDCCCXXXIV.

☞ *An eloquent and classical Oration.*

23. The Cyclopædia of Practical Medicine and Surgery; a Digest of Medical Literature. Edited by ISAAC HAYS, M.D. Part I. Philadelphia, July, 1833.

24. The American Journal of the Medical Sciences. Nos. XXVI. and XXVII. For February and May, 1834.

25. A Demonstration of the Nerves of the Human Body. By JOSEPH SWAN. Quarto, pp. 82. Plates, XXV.

☞ *A notice of this in the Periscope.*

26. Memoir of the Life and Medical Opinions of JOHN ARMSTRONG, M.D. Formerly Physician to the Fever Institution of London, &c. To which is added, an Inquiry into the Facts connected with those Forms of Fever attributed to Malaria or Marsh Effluvium. By FRANCIS BOOTT, M.D. &c. In Two Volumes. Vol. II. Octavo, pp. 752. London, 1834.

27. The Principles and Practice of Obstetric Medicine, &c. By DAVID D. DAVIS, M.D. &c. Parts XXX. to XXXV. inclusive. Price 2s. each part.

☞ *To be noticed.*

28. Ossa Humana. Plate V. Price 5s.

☞ *The object of the Author of these Plates, Mr. Cummin, a pupil of St. George's Hospital, is to display the Anatomy of the Human Bones, in a series of lithographic plates. There will be four parts, each at the price of five shillings. This is the third. The lithography is pretty.*

29. The Dublin Practice of Midwifery. By HENRY MAUNSELL, M.D. &c. 12mo, pp. 244. London, 1834.

30. A Practical Treatise on Lepra Vulgaris, &c. and on some of the Local Varieties of Psoriasis. By EDWARD BECK, M.D. Ipswich. Octavo, pp. 74. 1834.

☞ *Noticed in the Periscope.*

31. A Treatise on that Affection of the Joints denominated Arthritis or Gout. By LEWIS LEESE, M.D. M.R.C.S. &c. Octavo, Stitched, pp. 48. London, 1834.

32. The Substance of a Lecture delivered at the Re-opening of the School founded by the late Joshua Brooks, Esq. By THOMAS KING, M.D. &c. Octavo, stitched, pp. 31. London, 1834.

☞ *A well-timed and well-written discourse, much superior to the generality of "Introductories."*

33. A Treatise, Practical and Theoretical, on the Nature of Cholera, with an Examination of the Moral and Physical Influence of the Doctrine of Contagion. By STEPHEN BROUGHAM, M.R.C.S.L. Falmouth, &c. Small 8vo. pp. 111. London, 1834.

### LITHOTRITY.

Mr. COSTELLO has been successful in comminuting a stone of extraordinary dimensions in his own institution. The calculus, when held in the instrument, measured three inches and a quarter in its long axis, and two inches three quarters in its smallest diameter. It was of fawn-coloured lithate of ammonia and hard. It was computed that one-third of the fragments was lost, as the patient collected only those which he voided during the night and morning—the rest of the day he generally spent walking about town. The portion collected was weighed at Savory's, Bond Street, and amounted to five ounces two drachms. If to this be added the weight of the third computed to be lost, we shall have a concretion weighing seven ounces, removed by lithotrity from a living man. This is certainly a splendid result. Mr. Costello stated, that had he not devised the double slide percussor, which has no limit to its grasping power, it would have been vain to attempt seizing such a mass by any other instrument. He ridiculed the idea of reducing not only this stone, but one of any considerable size and compactness by screw force. The patient's age was sixty-four, and he had laboured under his complaint upwards of five and twenty years.

### INDEX.

A.

Abdomen, wounds of the .......... 299
Abortions, manual assistance in .... 448
Abscess, mammary, treatment of.... 541
Abscesses in the iliac fossa ......... 328
Absentee, the, by Dr. James Johnson, 154
Abstinence, the curative effects of .. 498
Aconite, use of, in neuralgia........ 221
Aloes, medicinal properties of ..... 212
Anatomical plates, by Mr. Quain, &c. 184
Anatomy, pathological, Drs. Hope and Carswell on................ 184, 424
Aneurism, caused by a sequestrum of bone......................... 261
Aneurisms, internal, Mr. Harrison on, 445
Aneurisms, internal, Dr. Stokes on .. 459
Anus, fissures of the .............. 326
Apoplexy, pathology of............ 429
Arnott, Mr. his reply to Dr. Wise .. 287
Artery, iliac, Mr. Guthrie's operation on 478
Ascites, chronic, spontaneous cure of, 560
Asphyxia, Dr. Kay's treatise on .... 92
Asphyxia, Dr. Edwards on ........ 99
Asphyxia, treatment of ............ 103
Association, the Provincial Medical, Transactions of ................ 171

B.

Barytes, poisoning from............ 548
Beck, Dr. his essay on lepra ........ 495
Belden, Dr. report of Jane Rider's case, 440
Bell, Sir C. on wounds of the knee-joint......................... 275
Bellingeri, his inaugural dissertation on the 5th and 7th pairs of nerves. 413
Bellingeri, his case of opisthotonos .. 503
Beobachtungen, Von Seiler on ...... 49
Bichat, his views on asphyxia ...... 93
Blackley, Dr. on the pulse.......... 433
Blood, effusion of, within pericardium, 147
Blood, Dr. Carson on the motion of, 175
Blood, on the influence of gravity on, 204
Bodies, living, effects of mercury upon, 213
Bones, cysts developed in substance of, 302
Botany, medical, Drs. Graves and Morries on .................... 106
Botany, medical, Messrs. Stephenson and Churchill on ............... 106
Brain, disorder of, after an accident.. 262
Brain, pathological anatomy of the .. 425
Brodie, Mr. on diseases of the joints.. 1
Brodie, Mr. on inflamed synovial membrane......................... 3
Brodie, Mr. on ulceration of cartilages, 16
Brodie, Mr. on ulceration of synovial membrane .................... 12
Brodie, Mr. on morbid alteration of synovial membrane.............. 13
Brodie, Mr. on caries of the spine.... 331
Brodie, Mr. on tumors and loose cartilages in the joints ............. 341
Brodie, Mr. on malignant diseases of the joints ..................... 343
Brodie, Mr. on hysterical affections of the joints ..................... 345
Brodie, Mr. on gouty alteration of the joints ....................... 348
Brodie, Mr. on inflammation of the bursæ mucosæ ................ 349
Brodie, Mr. on ununited fractures .. 529
Bronchotomy, case of ............ 263

C.

Cancerous diseases, treatment of .... 518
Carbutt, Dr. his clinical lectures..... 563
Carbutt, Dr. on inflammatory diseases 565
Caries, Mr. Wickham on .......... 71
Caries of the spine, Mr. Brodie on .. 331
Carson, Dr. on hæmorrhage within the pericardium ................ 147
Carson, on the motion of the blood .. 175
Carswell, Dr. his morbid anatomy . .. 42
Carswell, Dr. on softening from inflammation........................ 43
Carswell, Dr. on softening from arterial obliteration .............. 47
Carswell, Dr. on softening from gastric juice ...................... 47
Carswell, Dr. on hæmorrhage ...... 430
Cartilages, on ulceration of the...... 16
Cartilages, loose, in the joints ...... 341
Case of protracted somnolency ...... 217
Case, extraordinary, of paraplegia.... 228
Case of apparent death .......... 229
Cataract, Mr. Guthrie on extraction of, 178
Catarrhal ophthalmia, Mr. Tyrrell on, 454
Cauteries, M. Dupuytren on........ 327
Cayol, Dr. on cancerous diseases .... 517
Cellular membrane of joints, diseases of 76
Cerebellum, sympathy of, with the generative organs ............... 212
Cerebellum, curious disease of the .. 501
Cerebral diseases, Dr. Stokes' lectures, 542
Chemistry, organic, M. Raspail on .. 365
Chitty, Mr. his medical jurisprudence, 462
Chloruret of lime in blenorrhagia.... 510
Cholera in Ireland ................ 150
Cholera, nitrate of silver in ...... .. 444

Cholera, editorial remarks on the.... 484
Cholera, saline emetics in .......... 487
Chord, spinal, diseases of the........ 375
Circulation of the nervous system, Mr. Earle on ...................... 143
Circulation visible in some cryptogamous plants .................. 368
Circulation, Mr. Handley on........ 390
Climate of Penzance .............. 468
Clinical reports, from the Meath Hosp. 260
Clinical reports from Glasgow Hosp. 265
Clinical reports, from the Birmingham Eye Infirmary.................. 271
Clinical reports, from the Middlesex Hospital ...................... 275
Clinical report from the Limerick Infirmary ...................... 550
Clinical observations, by Dr. Graves.. 557
Clinique Chirurgicale of M. Dupuytren 289
Clitoris, chronic enlargement of .... 489
Club-foot, M. Dupuytren on the .... 322
Club-foot, section of tendo-Achillis in, 510
College of Surgeons, last curriculum of 569
Combe, Dr. A. on health & education, 168
Concours, remarks on the French.... 283
Condylomata, venereal, Mr. H. Johnson on.......................... 241
Congenital dislocation of the femur.. 320
Consumption curable, Dr. Ramadge on 62
Cooper, Mr. B. on fractures........ 255
Creosote, use of, in ulcerations...... 221
Creosote, use of, in ulcerations...... 505
Creosote, observations on .......... 527
Crepitatio musculorum, case of...... 452
Croton oil, external use of.......... 235
Curtis, Mr. essay on deaf and dumb .. 495
Cyanosis, interesting case of........ 234
Cyanuret of potass, death caused by, 222
Cysts, developed in the substances of bones .......................... 302
Cysts, serous, M. Dupuytren on .... 306

D.

Deaf and dumb, Mr. Curtis essay on, 495
Deafness, Mr. Swan on ............ 380
Death, case of apparent............ 229
Death, Dr. W. Philip on .......... 362
De Caudolle, his vegetable physiology, 353
Deformities, spinal, infirmary for. .. 282
Diabetes insipidus, case of.......... 563
Diagnosis, Dr. M. Hall's principles of, 493
Dickenson, Mr. his translation of Rayer 391
Dictionary of terms, by Dr. S. Palmer, 477
Digitalis, clinical observations on the use of .......................... 201
Disease, mesenteric, interesting case of 226
Diseases of the thymus gland, on the.. 195
Diseases of skin, Rayer's treatise on.. 391
Dislocation of hip into foramen ovale.. 250
Dislocation of the lens ............ 273
Dislocations of humerus, reduction of, 178
Dispensary, self-supporting ........ 230
Dispensary, London North-west Self-supporting .................... 567
Dropsy, cured after tapping ........ 167
Dropsy cured after paracentesis...... 251
Dropsy, albuminous urine in........ 562
Dupuytren's, M. clinique chirurgicale, 289
Dupuytren, M. on gun-shot wounds.. 291
Dupuytren, M. on injuries of the head, 296
Dupuytren, M. on wounds of the chest, 298
Dupuytren, M. on cysts within bones, 302
Dupuytren, M. on serous cysts...... 306
Dupuytren, M. on the nail growing into the flesh ...................... 309
Dupuytren, M. on dislocations of the humerus ........................ 312
Dupuytren, M. on congenital dislocation of the femur ................ 320
Dupuytren, M. on the club-foot .... 322
Dupuytren, M. on laceration of the perinæum in females ............ 323
Dupuytren, M. on fissures of the anus, 326
Dupuytren, M. on cauteries & moxas, 327
Dupuytren, M. on abscesses in the iliac fossa .................... 328
Dupuytren on spontaneous gangrene, 512
Dzondi, M. on morbus coxarius .... 238

E.

Earle, Mr. I. W. on functions of nerves 116
Eccles, Mr. on ulcers of the leg...... 157
Editorial remarks on the cholera .... 484
Education, Dr. A. Combe on........ 168
Edwards, Dr. experiments on suspended respiration ................ 99
Egyptian mummies, Mr. Pettigrew on, 84
Elementary forms of disease, illustrations of ...................... 42
Elongation of the limb in hip-disease considered .................... 80
Emetics, saline, in cholera ........ 487
Empyema, case of ................ 253
Encysted tumor on the neck ........ 225
Epigastric pulsations, Dr. Stokes on.. 448
Epilepsy, treatment of ............ 214
Epistaxis, on the use of cold in...... 212
Ergot, danger of large doses of the .. 435
Eruptions, syphilitic, Mr. H. J. Johnson on.......................... 244
Erysipelas, Dr. Macfarlane on ...... 266
Erysipelas, different opinions as to the treatment of .................. 267
Erysipelas, Mr. Arnott on.......... 278
Erysipelas, symmetrical, Dr. Graves on, 559
Everest, Mr. on homœopathy ...... 439
Exanthemata, M. Rayer on the...... 398
Exostosis, Mr. Wickham on ........ 69
External use of croton oil .......... 235
Eyelids, tumors of the ............ 275
Eyes, absence of the .............. 49
Eyes, particular malformations of the, 55

F.

Females, certain nervous affections in, 165
Femur, fracture of the neck of the .. 166
Femur, congenital dislocation of the.. 320
Fever in the horse ................ 91
Finger, adhesion of a separated...... 228
Fissure of the anus, M. Dupuytren on, 326
Fœtus, formation of the omenta in the, 222
Forbes, Dr. on climate of Penzance .. 468
Foreign body found in the heart .... 177
Fracture of the skull, case of........ 483
Fracture, compound, cases of ...... 553
Fractures of the ribs and sternum .. 255
Fractures, ununited, Mr. Brodie on.. 529
Fragmenta de viribus medicamentorum 492

G.

Ganglion, otic, account of the ...... 465
Gangrene, case of spontaneous dry .. 512
Generative organs, sympathy of cerebellum with ................. 212
Geology of Switzerland..... .... .. 155
Gouty alteration of the joints ... .. 348
Graves, Dr. his Hortus Medicus .... 106
Graves, Dr. his clinical observations.. 557
Graves, Dr. on pneumo-thorax..... 558
Graves, Dr. on the administration of calomel ..................... 559
Graves, Dr. on diffuse inflammation.. 560
Graves, Dr. on albuminous urine in dropsy...................... 562
Gravity, the influence of, on the blood, 204
Gun-shot wounds, M. Dupuytren on.. 291
Guthrie, Mr. on extraction of cataract, 178
Guthrie, Mr. his operation of tying the common iliac artery ............ 478

H.

Hæmoptysis, on the application of cold in..................... .. 212
Hæmorrhage, Dr. Carswell on ...... 430
Hæmorrhagic tendency in a family .. 232
Hall's, Dr. M. principles of diagnosis, 493
Hall, Dr. M. on the reflex function of the medulla oblongata, &c........ 116
Handley, M. on the circulation...... 390
Hangsted, on the thymus gland .... 195
Harrison, Prof. on internal aneurisms, 445
Hawkins, Mr. on the urinary organs.. 530
Hawkins, Mr. on hernia .......... 533
Health, Dr. A. Combe on .......... 168
Hearing, relations of the cranium with 240
Hearing, disordered states of........ 380
Heart, foreign body found in the .... 177
Heart, Majendie on the sounds of the 208
Heat, animal, source of............ 209
Heat, morbid development of ...... 226
Hemiplegia, case of .............. 225
Henderson, Dr. his translation of Raspail ......................... 365
Hip-disease, Mr. Wickham on ...... 80
Hip-disease, on lengthening & shortening of the limb in ............ 80
Hip-disease, dislocation from ...... 83
Homœopathy, Mr. Everest on ...... 439
Homœopathy, Dr. Quin's work on .. 492
Homoiopathism, failure of, in Russia.. 223
Hooping-cough, M. Blache on ...... 210
Hope's, Dr. pathological anatomy, 184, 424
Hope, Dr. on diseases of the uterus .. 184
Hope, Dr. on diseases of the brain .. 425
Horse, Mr. Percival on diseases of the 88
Hospitals, provincial, memorial from 161
Humerus, dislocated, mode of reducing the. ..................... 178
Humerus, dislocations of the........ 312
Hydatids of kidney, passed by urethra, 453
Hydatids, uterine, case of .......... 176
Hydriodate of iron, Dr. A. T. Thomson on...................... 476
Hydro-bronchocele, case of.... 513, 554
Hydrophobia, case of.............. 173
Hypertrophy of the mammæ........ 528
Hypertrophy of the stomach........ 502
Hysterical affections of the joints .... 345

I.

Iliac artery, Mr. Mott's operation of tying the...................... 483
Infants, pulmonary organic disease in, 230
Infants, purulent ophthalmia in .... 239
Inflammation, chronic of the prepuce, 249
Inflammation, diffuse, Dr. Graves on, 560
Inflammation of synovial membranes, 3
Inflammation of the bursæ mucosæ .. 349
Inflammation of the nerves ........ 122
Inflammation of the ovum.......... 502
Inflammation, softening from; description ...................... 43
Injections of cold water into the umbilical cord ................... 230
Insanity, ceasing after the operation of trephining .................... 262
Insanity, Dr. Mayo on ............ 466
Intestines, sutures in wounds of .... 299
Iodine, on the exhibition of ........ 436
Iodine, use of, in scrofula .......... 216
Ioduret of iron, Dr. A. T. Thomson on, 476
Ireland, cholera in ............... 150

J.

Johnson, Dr. James, description of an absentee ..................... 154
Johnson, Mr. H. J. on venereal warts, &c. 241
Johnson, Dr. singular case of crepitatio musculorum ............... 452
Joint, knee, wounds of ............ 275
Joints, Mr. Brodie on diseases of the, 1
Joints, inflamed synovial membrane of 3
Joints, ulcerated synovial membrane of 12
Joints, altered synovial membrane of, 13
Joints, ulceration of cartilages of.... 16

Joints, scrofulous disease of ........ 33
Joints, Mr. Wickham's treatise on the 68
Joints, loose cartilages in the ....... 341
Joints, malignant diseases of the .... 343
Joints, hysterical affections of the.... 345
Joints, gouty alteration of the ...... 348
Jurisprudence, medical, Mr. Chitty's work on ..................... 462

K.

Kane, Mr. his surgical report ....... 550
Kay, Dr. treatise on asphyxia ...... 92
Kidney, tuberculous affection of the.. 174
Kidney, cysts in the .............. 186
Kidney, hydatids of the, voided by the urethra ....................... 453

L.

Laceration of centre of the perineum, 323
Lead, paralysis from .............. 514
Lecturers, circular to.... ......... 570
Lectures, Dr. Stokes', on cerebral diseases ........................ 542
Leeches, preservation of .......... 229
Lens, dislocation of the............ 273
Lepra, Dr. Beck's essay on ......... 495
Leprosy of the jaws, Dr. Shapter on, 111
Literary curiosities................ 515
Liverpool Medical Society, transactions of the.................... 171
Lumbricus found in a wound ....... 215
Lung, case of laceration of the ..... 257
Lungs, spurious melanosis in the .... 166
Lymphatics of the arm, disease of the, 264

M.

Macfarlane, Dr. his clinical reports .. 266
Majendie on the sounds of the heart.. 208
Malaria, Dr. Weatherhead on ...... 157
Malformations, &c. of the eyes, Von Seiler on ...................... 49
Mammæ, hypertrophy of the ...... 528
Mammary abscess, treatment of .... 541
Mayo, Dr. his essay on insanity .... 466
Medica sacra, Dr. Shapter's treatise on 109
Medical reform, remarks on ........ 283
Medical jurisprudence, Mr. Chitty's work on ..................... 462
Medical schools, provincial, Dr. Collins on.......................... 571
Melanosis, spurious, of the lungs .... 166
Menorrhagia, use of steel in ........ 209
Mental affection, anomalous case of.. 147
Mental illusions, cases of .......... 525
Mercury, effects of, on living bodies.. 213
Mercury, use of, in rheumatism .... 214
Mesenteric disease, interesting case of, 226
Middlemore, Mr. his clinical reports.. 271
Monomania, homicidal ............. 210
Monstrosities, Velpeau's remarks on, 208
Morals, relation of, to insanity ..... 466
Morbid alteration of synovial membrane ........................ 13
Morbus coxarius, shortening of the limb in.......................... 238
Morbus oryzeus ................... 471
Motory functions, curious disturbance of the ....... ............... 501
Mott, Mr. his reclamation.......... 483
Moxas, M. Dupuytren on .......... 327
Mummies, Egyptian, Mr. Pettigrew on 84

N.

Nails growing into the flesh, on...... 309
Nerves, diseases of, Mr. Swan on.... 116
Nerves, functions of, Mr. Earle on .. 116
Nerves, inflammation of the........ 122
Nerves, ulceration of the .......... 123
Nerves, division of the ............ 125
Nerves, spinal, the diseases of the.... 371
Nerves, sensual, the diseases of the .. 379
Nerves, sympathetic .............. 384
Nerves, Bellingeri on the fifth and seventh pairs .................. 413
Nerves, Mr. Swan's demonstration of, 494
Nervous system, reflex function of, Dr. Hall on the..................... 116
Nervous affections in females ....... 165
Neuralgia, simple treatment of....... 216
Neuralgia, use of aconite in ........ 221
Neuralgia, Scarpa's observations on.. 227
Neuroses, Dr. Stokes' remarks on the, 543
Nitrate of silver in cholera ......... 444
Nosology, Dr. Weatherhead's synopsis 145

O.

O'Beirne on hydrocele of the neck .. 554
Operation, Sigaultian, case of ...... 500
Ophthalmia, purulent, in infants .... 239
Ophthalmia treated with sublimate lotions......................... 509
Ophthalmia, catarrhal, Mr. Tyrell on, 454
Opisthotonos, Bellingeri's case of.... 503
Omenta, formation of, in the fœtus.. 222
Organic chemistry, M. Raspail's .... 365
Organs, urinary, Mr. Hawkins on the, 530
Osseous cysts, M. Dupuytren on .... 302
Otic ganglion, account of the ....... 465
Ovaritis puerperalis, cases of ...... 232
Ovum, inflammation of the membranes of........................ 502

P.

Palmer, Dr. S. his dictionary........ 477
Pancras Street self-supporting dispensary .......... ........... 280
Paracentesis thoracis, case of ...... 253
Paraplegia, extraordinary case of .. . 228
Pelviotomy, case of .............. 500
Penzance, the climate of .......... 468
Percival, Mr. on the diseases of the horse ........................... 88

Perforation of strictures ........... 473
Pericardium, effusion of blood within the ........................... 147
Perineum, laceration of the centre of, 323
Periodicals, foreign, spirit of the .... 193
Pertussis, remarks on ............. 210
Pertussis, Mr. Sandwith on ........ 471
Pettigrew, Mr. on Egyptian mummies, 84
Philip, Dr. W. on sleep and death.... 359
Phlebitis, Mr. Arnott on .......... 287
Phrenological jurisprudence ........ 210
Physiology, vegetable, De Caudolle on. 353
Placenta, new method of detaching the 230
Plants, cryptogamous, the circulation in some ...................... 368
Pneumo-thorax, case of ........... 259
Pneumo-thorax, Dr. Graves' case of.. 558
Poisoning from sulphuric acid .. ... 490
Poisoning from oxalic acid ......... 490
Poisoning from corrosive sublimate.. 491
Poisoning by barytes, and by the mineral acids ..................... 548
Poisons, Dr. Roupell's work on ..... 489
Pomegranate bark against tænia .... 213
Pontine marshes, Dr. Weatherhead on 152
Porrigo, M. Biett's treatment of .. . 232
Porrigo, the varieties of............ 406
Portio dura; is it a nerve of hearing? 382
Portio dura, Bellingeri on the ...... 418
Prepuce, chronic inflammation of the, 249
Provincial hospitals, memorial from.. 161
Provincial medical schools, Dr. Collins on......................... 571
Puerperal ovaritis, cases of ........ 232
Pulse, the influence of position on the 433

Q.

Quin, Dr. his work on homœopathy, 492

R.

Ramadge, Dr. his consumption curable 62
Raspail, M. his organic chemistry.... 365
Raspail, M. on muscular contraction, 367
Raspail, M. on the visible circulation in some cryptogamous plants .... 368
Rayer, M. on the diseases of the skin, 391
Rayer, M. his classification ..... ... 392
Rayer, M. on the varieties of vaccinella 404
Recess, the, by Dr. James Johnson .. 154
Reclamation of Mr. Mott .......... 483
Recto-vesical lithotomy, case of .... 172
Reflex function of the medulla oblongata, &c...................... 116
Reform, medical, remarks on ...... 283
Respiration, effects of imperfect .... 230
Retroversion of the uterus.......... 212
Rey, M. his treatise on the cerebro-spinal axis ..................... 225
Rheumatism, use of mercury in .... 214
Ribs, fractures of ................ 255
Rice, diseases caused by damaged.... 472
Rider, Jane, case of .............. 440
Roupell, Dr. his work on poisons.... 489
Rupture of uterus ................ 215
Rupture of the vagina during delivery 224
Russia, failure of homoiopathism in.. 223

S.

Sacred writings, on the diseases mentioned in....................... 109
Salt, emetics of, in cholera.......... 487
Sclerotitis rheumatica, Mr. Middlemore on ...................... 273
Scrofula, treatment of, in Germany.. 211
Scrofula, use of iodine in .......... 216
Scrofula, Dr. Eager on ............ 435
Scrofulous disease of joints ........ 33
Secale cornutum, history of effects of 106
Seiler on malformations of eyes...... 49
Self-supporting dispensary ........ 280
Shapter, Dr. his sacra medica ...... 109
Sickness, sweating, M. Rayer on the 403
Sirocco, Dr. Weatherhead on the . .. 155
Skin, M. Rayer on diseases of the.... 391
Sleep, Dr. W. Philip on.. ......... 359
Societies, temperance, in America ... 159
Softening from inflammation; description ..................... 43
Softening from obliteration of arteries, 47
Softening from action of gastric juice, 47
Somnambulism, case of. .......... 440
Somnolency, extraordinary case of protracted.......................... 217
Soot, a substitute for creosote ...... 506
Spinal deformities, infirmary for .... 282
Spine, Mr. Brodie on caries of the.... 331
Spots of blood, semen, etc. on linen.. 516
Stafford, Mr. on the perforation of strictures....................... 473
Staphyloma, Mr. Middlemore on .... 272
Staphyloraphy, description of ...... 497
Steel, use of, in menorrhagia ...... 209
Sternum, case of fracture of........ 258
Stokes, Dr. on internal aneurisms .. 458
Stokes', Dr. lectures on cerebral diseases ....................... 542
Stokes, Dr. on epigastric pulsations.. 448
Stomach, hypertrophied, case of .... 502
Strictures, treatment of, by perforation 473
Stromeger, Dr. on the club-foot .... 510
Suppression of the urinary and anal evacuations during eleven years .. 228
Surgeons, latest curriculum of College of.......................... 569
Sutures, in wounds of the intestines.. 299
Swan, Mr. on diseases of the nerves.. 116
Swan, Mr. on diseases of spinal nerves 371
Swan, Mr. on injuries of spinal chord 375
Swan, Mr. on diseases of sensual nerves 379
Swan, Mr. on the portio dura ...... 382
Swan, Mr. on diseases of the sympathetic nerve.................... 384

Swan, Mr. on tetanus ........... 386
Swan, Mr. his demonstration of nerves 494
Sympathy, examples of morbid...... 214
Synovial membrane, Mr. Brodie on inflammation of.................. 3
Synovial membrane, on ulceration of, 12
Synovial membrane, morbid change of 13
Syphilis, secondary, Mr. H. Johnson on 244

T.

Tænia, pomegranate bark against.... 213
Taliacotian union, case of.......... 228
Tarsal cartilages, disease of ...... . 272
Temperance societies in America.. .. 159
Tendo Achillis, section of the, in club-foot........................ 510
Tetanus, Mr. Swan on ............ 384
Thomson, Dr. A. T. on ioduret of iron 476
Thymus, description of its anatomy and pathology.................. 194
Transactions of the Provincial Medical Association ................ 171
Transactions of the Liverpool Medical Society ...................... 171
Trephine, use of, in caries and necrosis 75
Trephining, interesting case of...... 262
Trigeminus nerve, Bellingeri on the.. 413
Tuberculous affection of the kidneys 174
Tumor, encysted, case of .......... 225
Tumors of the eyelids ............ 275
Tumor, anomalous, in the hypochondrium....................... 438
Tuthill, Sir George, defence of the College of Physicians............ 113
Tyrrell, Mr. on catarrhal ophthalmia 454
Tytler, Dr. his views respecting rice.. 472

U.

Ulcers of the leg, Mr. Eccles on .... 157
Ulcers, use of creosote in .......... 221
Ulceration of the cartilages, Mr. Brodie on ....................... 16
Ulceration of the nerves, Mr. Swan on 123
Urine, carbonate of ammonia in the.. 561
Urinary organs, Mr. Hawkins on the 530
Uterine hydatids, case of .......... 176
Uterus, Dr. Hope on the ulcer of the 185
Uterus, retroversion of the ...... .. 212
Uterus, rupture and extreme tenuity of 215

V.

Vaccination in Italy, report on...... 193
Vaccinella, varieties of ............ 404
Vagina, rupture of the, during delivery 234
Vegetable physiology, De Caudolle on, 353
Velpeau, M. on certain monstrosities 208
Venereal warts, &c. Mr. H. Johnson on 241
Veratria, editorial report on ........ 163
Vindiciæ medicæ, by Sir G. Tuthill .. 113
Violation, suspected, case of........ 524

W.

Warts, venereal, Mr. H. Johnson on.. 241
Weatherhead, Dr. synopsis of nosology 145
Weatherhead, Dr. on Pontine marshes 152
Weatherhead, Dr. on the sirocco.... 155
Weatherhead, Dr. on malaria ...... 157
Wickham, Mr. on the joints........ 68
Wickham, Mr. on exostosis ........ 69
Wickham, Mr. on necrosis.......... 70
Wickham, Mr. on caries .......... 71
Wickham, Mr. on inflamed synovial membrane .................... 76
Wickham, Mr. on inflamed cellular membrane of joint ............... 76
Wickham, Mr. on relaxation of ligaments ........................ 78
Wickham, Mr. on abscess and anchylosis.......................... 79
Wickham, Mr. on disease of the hip.. 80
Williams, Dr. on asphyxia.......... 105
Wilson, Dr. on cholera in Ireland.... 150
Wounds of the knee-joint.......... 275
Wounds, gunshot, M. Dupuytren on 291

### LITERARY NOTICES.

In the Press,

The Principles of Ophthalmic Surgery, being an introduction to a knowledge of the Structure, Functions, and Diseases of the Eye, including the author's new views of the Physiology of the Organ of Vision. By John Walker, Assistant-Surgeon to the Manchester Eye Institution. Foolscap octavo.

The Surgeon's Practical Guide in Dressing and in the Methodic Application of Bandages. By Thomas Cutler, M.D. late Staff Surgeon in the Belgian Army. Foolscap octavo, illustrated by numerous engravings on wood.

